# A Self-Propagating Foaming Process of Porous Al-Ni Intermetallics Assisted by Combustion Reactions

**DOI:** 10.3390/ma2042360

**Published:** 2009-12-15

**Authors:** Makoto Kobashi, Naoyuki Kanetake

**Affiliations:** Graduate School of Engineering, Nagoya University, 1 Furo-cho, Chikusa-ku, Nagoya, Aichi 4648603, Japan; E-Mail: kanetake@numse.nagoya-u.ac.jp (N.K.)

**Keywords:** combustion foaming, Al_3_Ni, intermetallics, self-propagating high-temperature synthesis, exothermic reaction

## Abstract

The self-propagating foaming process of porous Al-Ni intermetallics was investigated. Aluminum and nickel powders were blended, and titanium and boron carbide powders were added as reactive exothermic agents. The blended powder was extruded to make a rod-shape precursor. Only one end of the rod precursor was heated to ignite the reaction. The reaction propagated spontaneously throughout the precursor. Pore formation took place at the same time as the reaction occurred. Adding the exothermic agent was effective to increase the porosity. Preheating the precursor before the ignition was also very effective to produce porous Al-Ni intermetallics with high porosity.

## 1. Introduction

Porous metals exhibit various unique physical and mechanical properties, such as low apparent density, low thermal conductivity, high specific stiffness, gas permeability and high strain energy absorbing capacity [1]. Porous aluminum [2,3,4,5,6,7] is one of the most enthusiastically investigated materials among a wide variety of known porous metals. However, there is a strong demand for practical usage of porous intermetallics or their composites that can endure severe environments such as high temperatures, highly corrosive atmospheres or high applied pressures [8,9,10]. In comparison with porous aluminum, the processing route of highly porous intermetallics has not been established yet. One problem for producing porous intermetallics is that the melting point of intermetallics is generally higher than that of light metals, and, therefore, it is more difficult and more energy consuming to produce large amount of pores in molten intermetallics. The authors have developed an innovative fabrication process for porous intermetallics using combustion synthesis, and reported porous Al-Ni intermetallics with porosities of more than 80% [11,12,13,14]. In this technique, porous Al-Ni intermetallics were fabricated by heating a reactive precursor consisting of aluminum, nickel, titanium and boron carbide (B_4_C) powders. Titanium and B_4_C powders were added as reactive exothermic agents, which increases the heat of reaction. When the precursor is heated, the exothermic reactions between (1) nickel and aluminum [15,16] and (2) titanium and B_4_C [17] shown below take place:

3Al + Ni → Al_3_Ni + 151 kJ/mole Ni (1)

3Ti + B_4_C → 2TiB_2_ + TiC + 761 kJ/ mole B_4_C (2)

**Figure 1:**
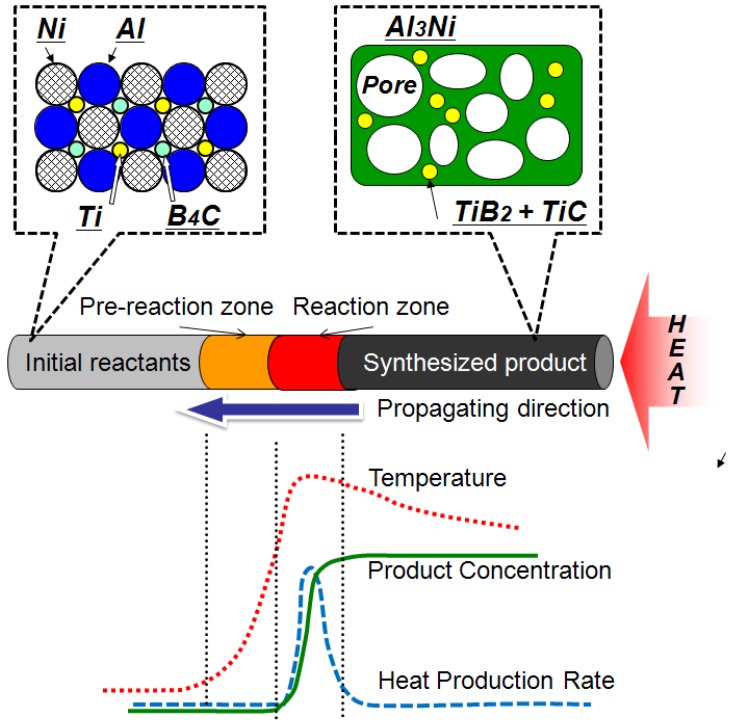
Concept of synthesizing porous Al-Ni intermetallics by the self-propagating foaming process.

The precursor expanded at the same time as the reaction due to evaporation of the gas phase (mainly hydrogen), which was originally adsorbed at or absorbed in the elemental powders [14]. In this work, an attempt was made to apply this combustion foaming technique to a self-propagating process. Figure 1 illustrates the brief concept of self-propagating foaming process.

**Figure 2:**
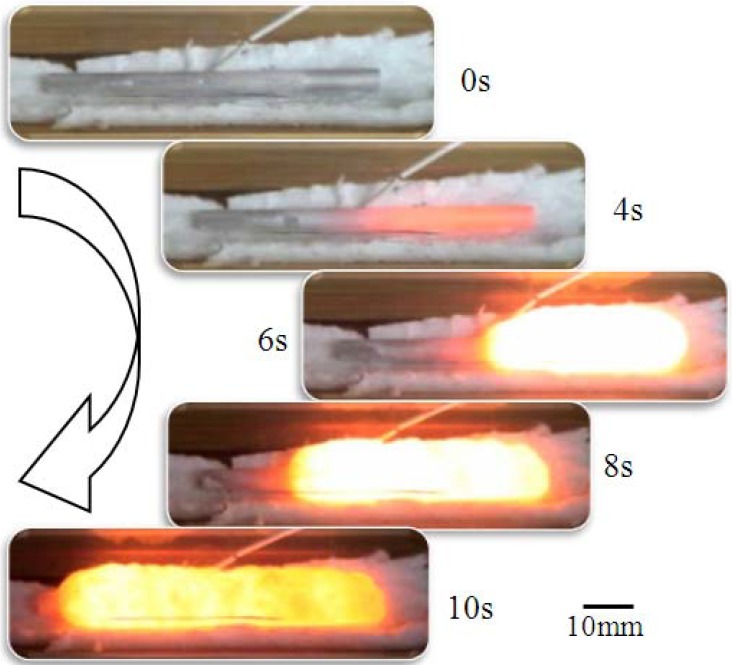
Self-propagation of the foaming process driven by the exothermic reaction (Al/Ni mole ratio:4.5, preheating temperature: 650 °C).

A long rod precursor made of Al, Ni, Ti and B_4_C powders was prepared by hot extrusion. When one end of the precursor (heated end) is heated and the exothermic reaction occurs, the heat of reaction preheats its neighboring zone (pre-reaction zone) and triggers the exothermic reaction again. Therefore, the reaction propagates spontaneously throughout the precursor from the heated end to the non-heated end. Figure 2 shows the typical self-propagation behavior of an extruded precursor. A rod-shape precursor is placed on ceramic wool in the air and was heated by induction coil from the right hand side of the figure. The self-propagating behavior of the reaction is clearly observed. The advantage of this process is that the energy to make a foam material is not necessarily supplied from the external source, but generated from the precursor itself (heat of reaction) [18,19]. This feature is quite helpful to fill a hollow component with a foam material. By the conventional foaming method using precursors, the precursor and the hollow component need to be heated together in a furnace, which sometimes gives an unfavorable thermal history to the hollow component. However, by the self-propagating technique, only a part of the precursor needs to be heated to ignite the reactive foaming. Once the reaction and foaming occur, they propagate throughout the precursor in the hollow component spontaneously. In the present paper, (i) the fundamental foaming behavior of Al-Ni precursors by a thermal explosion mode and (ii) effects of addition of a reactive exothermic agent and preheating treatment of the precursor on porosity and cell morphology by the self-propagating mode are discussed.

## 2. Experimental Procedure

### 2.1. Preparation of Precursor

**Figure 3:**
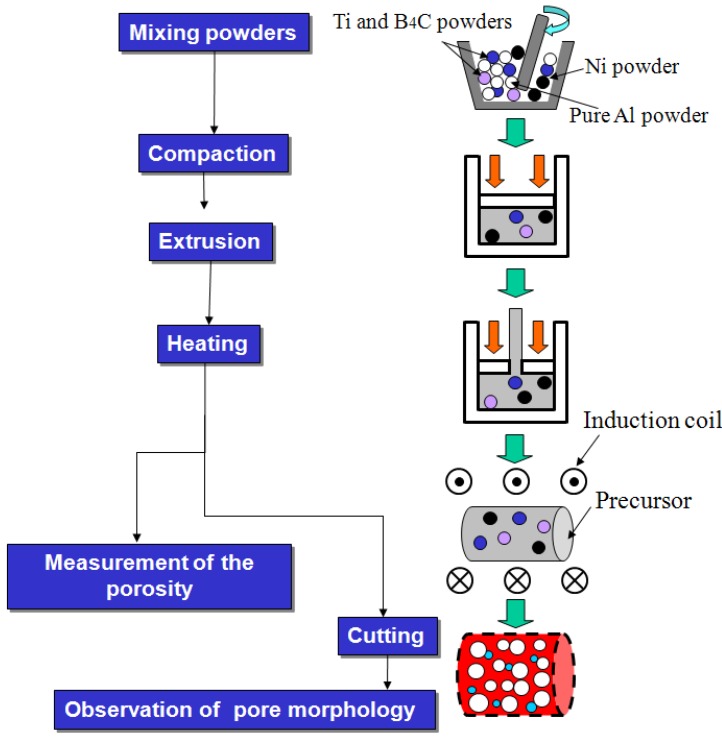
Flow chart of the experimental procedure.

Figure 3 shows the flow chart of the experimental procedure. The sizes of the starting material powders were Al (<44 μm), Ni (3–5 μm), Ti (<44 μm) and B_4_C (<10 μm), respectively. Aluminum and nickel powders were blended in appropriate molar blending ratios (Al/Ni mole ratio: 4.5). The foaming agent (Ti and B_4_C blended powder, Ti/B_4_C mole ratio: 3.0) was added at 0~20 vol%. The blended powder was then hot extruded at 500 °C to make rod-shaped precursors (ϕ 10 mm × L 150 mm).

### 2.2. Self-Propagating Blowing Process

**Figure 4:**
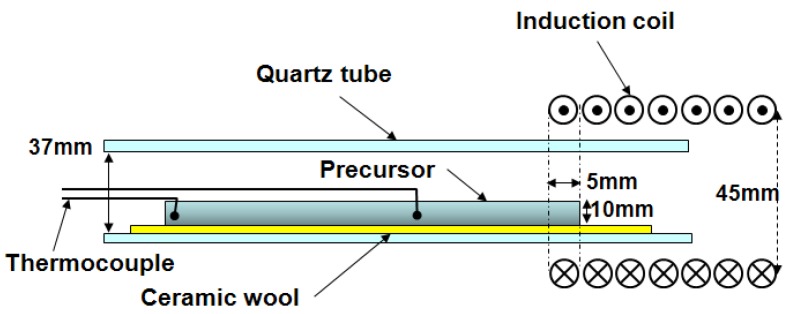
Schematic illustration of the induction heating apparatus.

The precursor was set in an induction heating apparatus as shown in Figure 4. The temperature of the specimen was measured by thermocouples (Pt/Pt-13%Rh, R type) directly embedded in the precursor. One end of the precursor was heated by an induction coil until the reaction started. The porosity was calculated from the measured density of the porous specimen by the Archimedes method and theoretical density of dense Al_3_Ti, Al, TiC, TiB_2_ composite of the same composition.

## 3. Results and Discussion

### 3.1. Fundamental Foaming Behavior of Al-Ni Precursor by Thermal Explosion Mode

**Figure 5:**
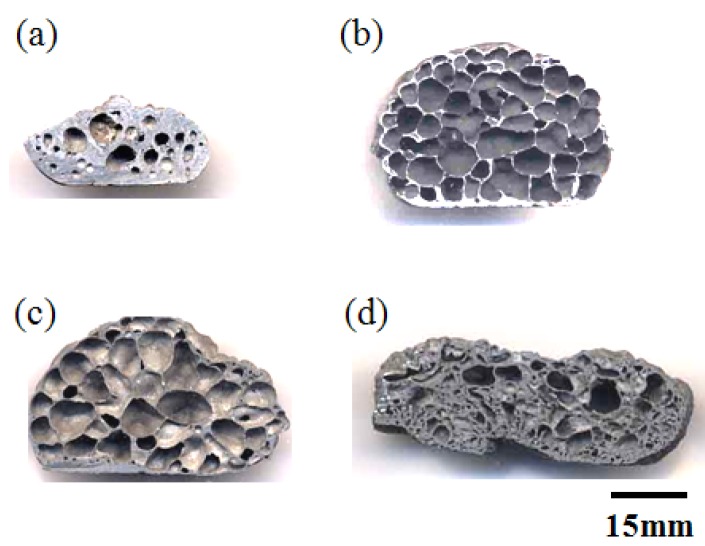
Cross-sections of porous Al-Ni intermetallics with different addition of foaming agent (Al/Ni = 4.5), (a) 0 vol%, (b) 5 vol%, (c) 10 vol%, (d) 20 vol%.

**Figure 6:**
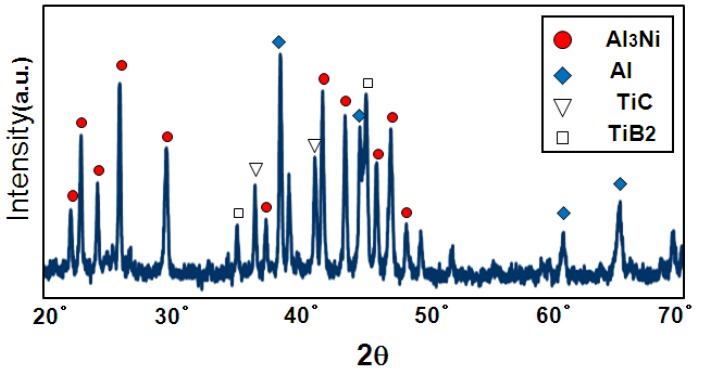
XRD analysis data of the specimen (Al/Ni blending ratio: 4.5, exothermic agent addition: 20 vol%).

**Figure 7:**
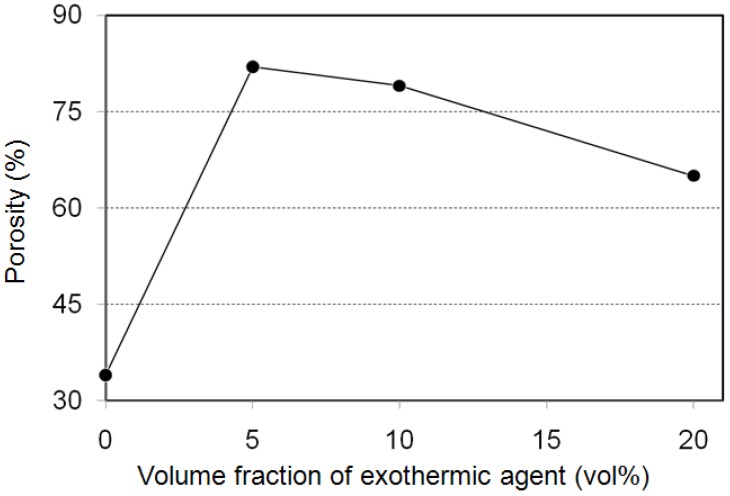
Porosity of porous Al-Ni intermetallics as a function of exothermic agent addition.

Prior to the self-propagating-mode process, the foaming behavior by a homogeneously heating the whole precursor in a furnace (thermal explosion mode) is discussed. Figure 5 shows macroscopic cross-sections of porous specimens fabricated by a thermal explosion mode from the cylindrical precursor (*ϕ* = 12 mm, *h* = 12 mm, Al/Ni mole ratio = 4.5, foaming agent = 0, 5.0, 10.0 and 20.0 vol%). As a result of the XRD analysis shown in Figure 6, the formation of Al_3_Ni, TiB_2_ and TiC as indicated in equations (1) and (2) can be confirmed. The porosity of the Al_3_Ni foam was attributed to gas release from the elemental powders. In the previous paper, we already reported that the main constituent of the gas in the pores was hydrogen, which was originally adsorbed at or absorbed in elemental powders [14]. Figure 7 shows porosity of the specimens. The specimens after the reaction showed high porosity by blending appropriate amount of foaming agent (5, 10 vol%). Porosity of the specimen with 5 vol% agent addition was 83%, whereas that of non-added specimen was 34%. This is because the foaming agent released substantial heat and assisted the progress of the reaction between aluminum and nickel, as indicated in the previous papers [11,12,13,14]. However, excessive addition (20 vol%) resulted in a collapsed cell morphology and low porosity. This may be related to the excessive increase in the combustion temperature. The viscosity of the specimen during combustion reaction was probably too low to maintain the porous structure when 20 vol% exothermic agent was added.

### 3.2. Effect of Reactive Exothermic Agent on Foaming Behavior by Self-Propagating Mode

**Figure 8:**
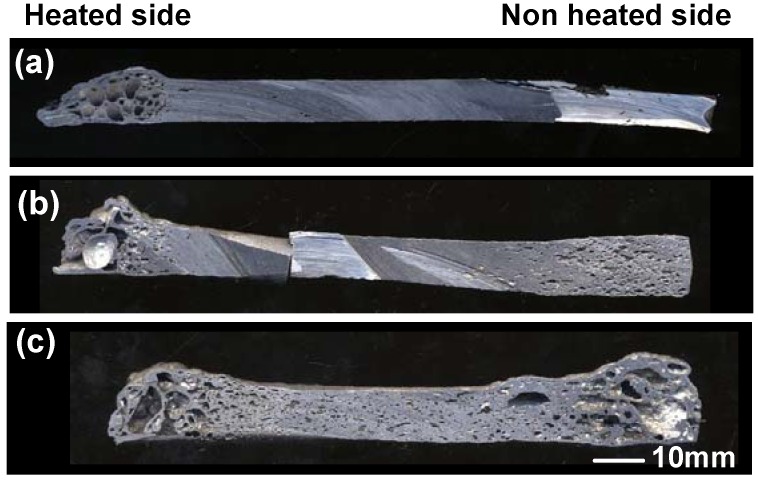
Cross-sections of specimens fabricated by self-propagating process (Al/Ni = 4.5), (a) without foaming agent, (b) 3 vol% and (c) 5 vol% foaming agent addition.

**Figure 9:**
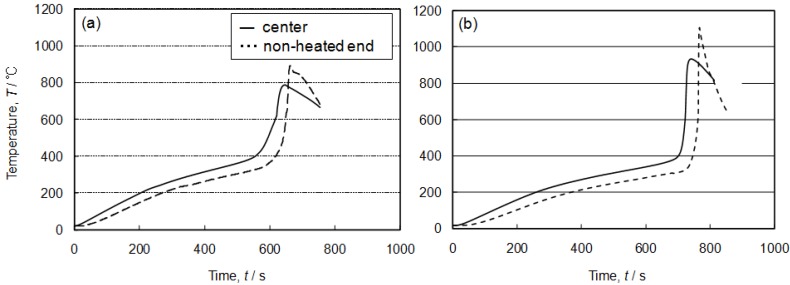
Temperature profile of the specimens at different measurement points, (a) without foaming agent addition and (b) 5 vol% foaming agent addition.

Figure 8 shows the cross-section of the specimens fabricated by the self-propagating process with different amounts of exothermic agent additions (0, 3, 5 vol%). The specimen without exothermic agent did not show any clear pore formation, except at the heated end. However, by adding exothermic agent by 5 vol%, pore formation was clearly confirmed. A relatively high porosity was achieved near both ends of the precursor (heated and non-heated ends), and the pore formation was not satisfactory at the center. Figure 9 shows temperature profile of both non-heated end and center part of the precursors. Without exothermic agent addition, the maximum temperature did not reach to the melting point of Al_3_Ti (854 °C) at the center [the solid line in Figure 9(a)]. However, by adding exothermic agent, the maximum temperature became higher than the melting point of Al_3_Ni. Hence, the addition of exothermic agent was effective for the self-propagating-mode foaming as well as the thermal-explosion-mode foaming process.

The maximum temperature of the non-heated end was higher than that of center. The heat of reaction at the reaction zone was transferred to its neighboring zone immediately because aluminum shows good thermal conductivity. However, the reaction heat at the non-heated end dissipated less quickly because the heat was transferred to the ambient atmosphere mainly by a radiation. This resulted in the higher maximum temperature at the non-heated end as shown by dotted lines in Figure 9.

### 3.3. Effect of Preheating

**Figure 10:**
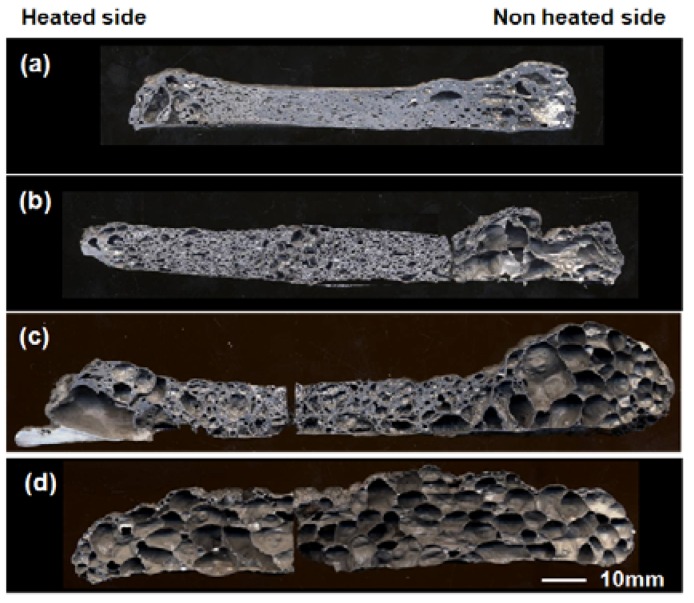
Cross section of specimens fabricated by self-propagating mode after preheating at various temperatures, (a) without preheating, (b) 200 °C, (c) 450 °C, (d) 650 °C.

**Figure 11:**
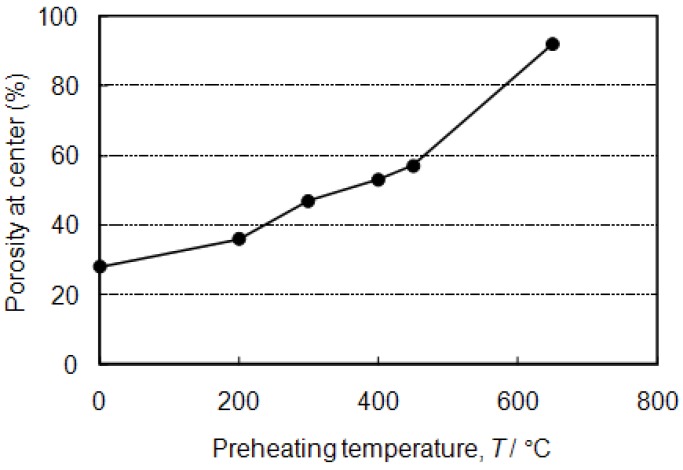
Porosity of the specimens at the center (50 mm in length) as a function of preheating temperatures.

Preheating treatment at various temperatures was carried out just before the ignition by induction heating. Figure 10 and Figure 11 show macroscopic cross-sections and the porosity of the specimens at the center part (50 mm in length) with 200~650 °C preheating. It is obvious that preheating treatment was effective to obtain high porosity, especially at 650 °C (just below the ignition temperature). By preheating at 650 °C, high porosity was achieved even at the center part, and a homogeneous porous structure was achieved.

## 4. Conclusions

A reactive foaming process of porous Al-Ni intermetallics was investigated. Aluminum and nickel powders were blended, and titanium and boron carbide powders were added as reactive exothermic agents. Porosity and pore morphology was examined by both the thermal explosion mode and self-propagating mode, and the following results were obtained.

1. With regards to the thermal explosion mode, the specimens after the reaction showed high porosity after blending appropriate amounts of foaming agent (5, 10 vol%). However, excessive addition (>20 vol%) resulted in the collapsed cell morphology and low porosity. This may be related to the high fraction of Al-Ni molten phase during the combustion reaction.
2. In the self-propagating mode, the specimen without exothermic agent did not show clear pore formation, except at the heated end. However, by adding 5 vol% of exothermic agent, pore formation was realized. Relatively high porosity was achieved near both ends of the precursor (heated and non-heated ends), but the pore formation was not satisfactory in the center. A preheating treatment was effective to obtain a high porosity. By preheating at 650 °C, high porosity was achieved, even in the center part, and a homogeneous porous structure was thus obtained.
